# Identification, Characterization and Antihypertensive Effect In Vivo of a Novel ACE-Inhibitory Heptapeptide from Defatted Areca Nut Kernel Globulin Hydrolysates

**DOI:** 10.3390/molecules26113308

**Published:** 2021-05-31

**Authors:** Xing Liu, Guanwen Li, Huimin Wang, Nan Qin, Lili Guo, Xiaomin Wang, Sang Shen

**Affiliations:** 1 College of Medicine and Food Engineering, Shanxi University of Chinese Medicine, Taiyuan 030619, China; sxzyydxlx@163.com (X.L.); sxzyydxlgw@163.com (G.L.); w1041252040@163.com (H.W.); cauguolili@sxtcm.edu.cn (L.G.); wangxiaomin061@126.com (X.W.); 2 Yunnan Institute of Food Safety, Kunming University of Science and Technology, Kunming 650500, China; sangshen@stu.kust.edu.cn

**Keywords:** areca nut kernel globulin, ACE-inhibitory peptide, endothelin-1, in silico screening, spontaneously hypertensive rats, molecular docking

## Abstract

The areca (*Areca catechu* L.) nut kernel (ANK) is a good potential protein source for its high protein content of 9.89–14.62 g/100 g and a high yield of around 300,000 tons per year in China. However, utilization of the areca nut kernel is limited. To expand the usage of ANK in pharmaceutical or foods industries, areca nut kernel globulin was extracted and angiotensin-I converting enzyme (ACE) inhibition peptides were prepared and identified using gel chromatography, reversed phase HPLC separation, UPLC-ESI-MS/MS analysis and in silico screening. Finally, a novel ACE-inhibitory heptapeptide (Ala–Pro–Lys–Ile–Glu–Glu–Val) was identified and chemically synthesized. The combination pattern between APKIEEV and ACE, and the inhibition kinetics, antihypertensive effect and endothlein-1 inhibition activity of APKIEEV were studied. The results of the molecular docking demonstrated that APKIEEV could bind to four active sites (not the key active sites) of ACE via short hydrogen bonds and demonstrated high ACE-inhibitory activity (IC_50_: 550.41 μmol/L). Moreover, APKIEEV exhibited a significantly lowering effect on both the systolic blood pressure and diastolic blood pressure of spontaneously hypertensive rats, and had considerable suppression ability on intracellular endothelin-1. These results highlight the potential usage of APKIEEV as ingredients of antihypertensive drugs or functional foods.

## 1. Introduction

Hypertension can induce serious diseases such as vascular sclerosis, stroke and coronary heart disease. About 10.8 million patients died of hypertension worldwide in 2019 [1]. Hypertension can be induced by many factors including heredity, obesity, stressful life and dietary habits [2]. The angiotensin I-converting enzyme (ACE) plays an important role in increasing blood-pressure through catalyzing the generation of angiotensin-II (a potent vasoconstrictor) and inactivating the bradykinin (a potent vasodilator) [3]. Moreover, the relationship between hypertension and excessive expression of endothelin-1 (ET-1) in the endothelium has been shed lighted by increasing studies [4]. Previous studies have demonstrated that ET-1 inhibition peptides could effectively decrease the systolic and diastolic blood pressure of spontaneously hypertensive rats [5,6]. Therefore, ACE inhibitors and ET-1 inhibitors are accepted as reasonable and practical therapy for hypertension. In recent decades, ACE-inhibitory peptides and ET-1 inhibition peptides derived from natural resource are given more attention because they do not have unacceptable side effects compared to synthetic drugs such as captopril [7]. The isolation, characterization, and relationship between the structure and bioactivity of ACE-inhibitory peptide derived from plant protein as well as animal and marine protein has been studied. It has been demonstrated that the activity of ACE-inhibitory peptide is dependent on the amino acid sequence, especially C-terminal tripeptide [8,9]. However, studies referring to ET-1 inhibition activity, in vivo bioavailability, action mechanism, and antihypertensive effect in vivo of ACE-inhibitory peptides are limited [3,7,8,9,10].

Areca (*Areca catechu* L.) is one of traditional Chinese medicines. Areca fruit (betel nut) is used to treat parasitic diseases and eliminate their accumulation in China [11]. The Hainan and Yunnan provinces are the main growth areas for areca in China. The areca nut kernel (ANK) is the main byproduct of the areca industry, and is rich in protein (9.89–14.62 g/100 g). The yield of the areca nut kernel is around 300,000 tons per year in China [12], suggesting that areca nut kernel is a potential protein resource. However, utilization of the areca nut kernel is limited until now. Most of the time, the areca nut kernel is directly discarded, which canl cause serious pollution problems. Although many studies have investigated the alkaloids, tannins, terpenes, pigment and fiber of the areca nut [13,14,15], little data on the areca nut kernel protein is available. Plant seed protein can be classified into five fractions: albumin, globulin, prolamine, glutelin-1 and glutelin-2 based on solubility. The globulin is a kind of protein fraction soluble in dilute salt solution and exists widely in cereal and oil crops such as soybean, coconut and oil palm [5,16]. The result of the pre-experiment of this study reveals that globulin is the major fraction of areca nut kernel, according for 49.37 g/100 g areca nut kernel protein. Moreover, the areca nut kernel globulin (ANKG) has the highest ACE inhibition activity (19.67% at 1.0 mg/mL) among areca nut kernel protein fractions, suggesting that ANKG is a potential source of ACE-inhibitory peptides. Thus, to expand the usage of ANK in pharmaceutical or foods industries, the identification, characterization and structure–activity relationship of ACE-inhibitory peptides from areca nut kernel globulin hydrolysates (ANKGH) were studied in the current study. The antihypertensive effect in vivo and ET-1 inhibition activity were investigated, too.

## 2. Results and Discussion

### 2.1. Purification of ACE-Inhibitory Peptides from ANKGH

**Figure 1:**
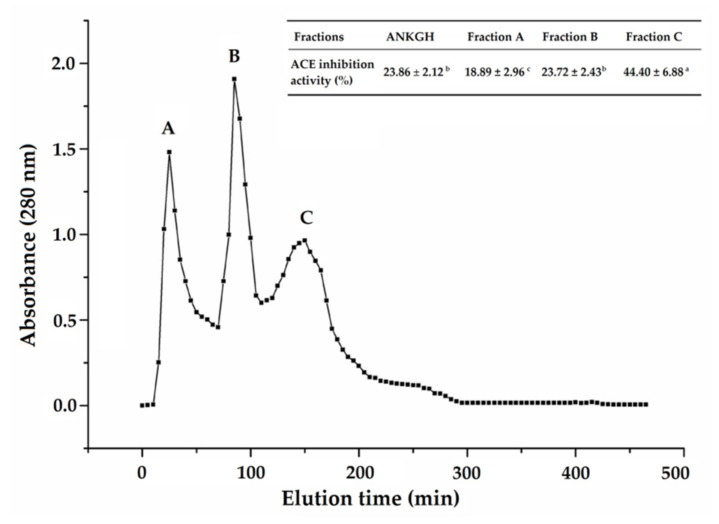
The separation profiles of areca nut kernel globulin hydrolysate (ANKGH) on Sephadex G-15 gel chromatography and angiotensin I-converting enzyme (ACE) inhibition activity of the subfractions. Uppercase letters (A–C) above the line and in the Table represent the subfractions. Different lowercase letters (^a–c^) in the Table mean significant difference (*p* < 0.05).

The extraction ratio of areca nut kernel globulin was 6.96 g/100 g defatted areca nut kernel. The cellulase used in the current study was to cause the collapse of the cell wall of the areca nut kernel, making more protein exposed. Both alcalase and trypsin were used to prepare ANKGH in the current study. The previous study demonstrated that the use of trypsin could improve the stability of produced peptides against gastrointestinal digestion [5]. The ACE-inhibitory activity and hydrolysis degree of ANKGH were 27.63% ± 1.33% and 13.67% ± 0.94%, respectively. This result demonstrates that enzymatic hydrolysis can improve the ACE-inhibitory of proteins. A similar trend was obtained by previous studies [4,5,6,9]. As shown in Figure 1, there were four fractions (A–C) collected after the purification of ANKGH with the Sephadex G-15 gel chromatography. The fraction C was chosen to further separation by RP-HPLC because of its highest ACE-inhibitory activity (44.40% ± 6.88%, at 1.0 mg/mL).

**Figure 2:**
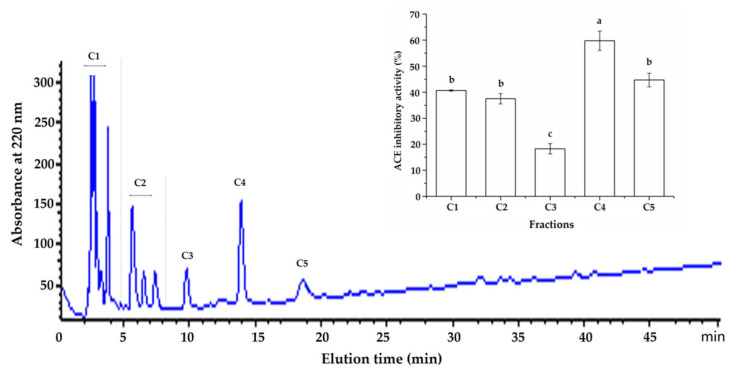
RP-HPLC profiles of fraction C and ACE inhibition activity of each subfraction. Different lowercase letters (a–c) above the bars indicate significant differences (*p* < 0.05).

The results in Figure 2 demonstrate that the fraction C was separated into five major subfractions (C1–C5) after the semi-preparative RP-HPLC purification. Because the subfraction C4 exhibited the highest ACE-inhibitory activity (59.76% ± 3.70% at 1.0 mg/mL) among these subfractions (C1–C5), it was selected and used for the peptide sequence analysis. In addition, previous studies have also demonstrated that Sephadex G-15 gel chromatography combined with RP-HPLC was an effective way for separation and purification of ACE-inhibitory peptides from protein hydrolysates [5,16].

### 2.2. Identification of Peptides from Subfraction C4 and In Silico Screening

**Figure 3:**
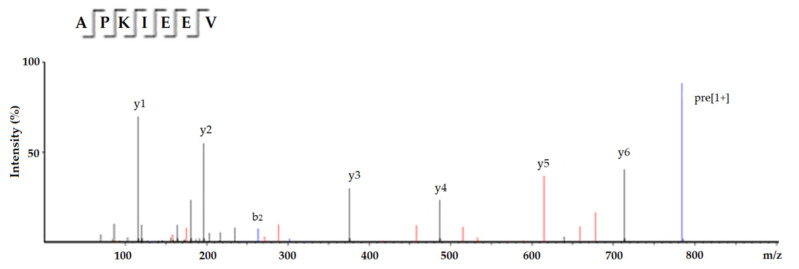
ESI-MS/MS spectrum of APKIEEV identified in areca nut kernel globulin hydrolysates.

**Figure 4:**
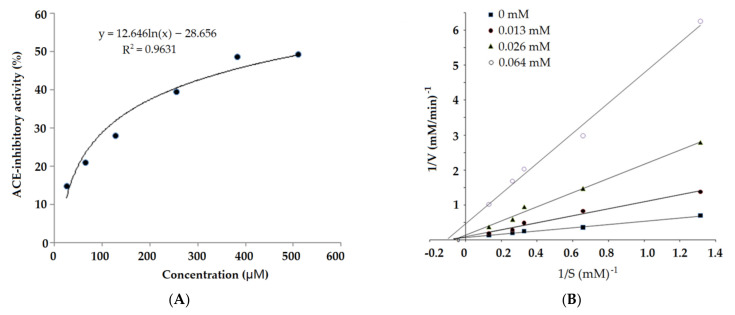
The ACE-inhibitory activity and the regression analysis (**A**), and Lineweaver–Burk plots of the ACE inhibition (**B**) of APKIEEV.

The results in Table 1 demonstrate that seven peptides sequence were identified in the subfraction C4 based on the fragment information and molecular mass calculated from the analysis of UPLC and ESI-MS/MS. After the in silico screening using the BIOPEP and AHTPDB databases, the screened result of the peptides and some predicted physicochemical properties were shown in Table 1. Only heptapeptide APKIEEV (784.99 Da) showed potential ACE-inhibitory activity and antihypertensive capacity, so it was chosen and chemically synthesized. The MS spectrum of APKIEEV is shown in Figure 3. As shown in Figure 4A, the regression equation of APKIEEV was y = 12.464 ln(x) − 28.656. The IC_50_ value of synthesized APKIEEV is calculated to be 550.41 μmol/L. Obviously, APKIEEV is rich in hydrophobic amino acids and has a Val residue in the C-terminal. Hydrophobic amino acids including Val are considered to play an important role in ACE-inhibitory activity of peptides [8]. The Glu residues in C-terminal tripeptide and branched Ile residue are also contributed to the activity of APKIEEV [3]. Moreover, the Pro residue in the N-terminal of APKIEEV is found to be responsible for increase in ACE-inhibitory activity of peptides [6]. Although ACE-inhibitory activity of peptides is negatively related with molecular weight [17], the IC_50_ value of APKIEEV (784.99 Da, IC_50_: 550.41) is much higher than (*p* < 0.05) that of NMAINPSKENLCSTFCK (1900.20 Da) identified in casein protein (IC_50_: 129.07 μmol/L) [18], the main reason may be that they have different combination patterns with ACE.

In addition, the hydrophilicity and amphiphilicity of APKIEEV were 0.74 and 0.89, respectively, suggesting that it is soluble in both aqueous solution and non-polar media [9,19]. These results demonstrate that APKIEEV has a relatively high ACE inhibition activity and can be used in both hydrophobic and hydrophilic systems [3].

### 2.3. ACE Inhibition Kinetics of Synthesized Peptides

As shown in Figure 4B, both the Michaelis–Menten constant (K_m_: −0.15) of ACE and the maximum velocity (V_max_: 14.22 mM/min) of the reaction decreased as concentration of APKIEEV increased, suggesting a uncompetitive inhibition modality. For uncompetitive inhibitors of ACE, they can inhibit ACE activity but cannot touch the central active site of ACE. The result means that APKIEEV can inhibit ACE activity through hindering the touch between ACE and its substrates, such as angiotensin-1 and bradykinin, but cannot bind to active sites of ACE [20]. Xu et al. [16] and Ling et al. [20] also identified uncompetitive inhibitors of ACE from soybean protein hydrolysates and tilapia skin gelatin hydrolysates, respectively. In addition, it has been found that competitive peptides always show a higher ACE-inhibitory activity than that of uncompetitive peptides [1,5].

### 2.4. Combination Pattern between ACE and Peptides

**Figure 5:**
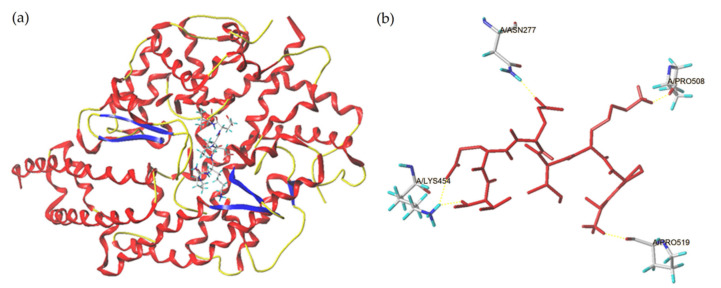
General overview (**a**) and local overview (**b**) of the best-ranked docking pose of APKIEEV binding with ACE (PDB: 1O8A).

The simulated combination pattern between peptide APKIEEV and ACE was shown in Figure 5a, and the best-ranked docking pose of APKIEEV binding with ACE was shown in Figure 5b. A previous study showed that ACE has three main active site pockets (S1, S2 and S1′). The S1 pocket includes Ala354, Glu384 and Tyr523 residues and the S2 pocket includes Gln281, His353, Lys511, His513 and Tyr520 residues, while the S1′ contains Glu162 residue [18]. APKIEEV can bind to four active sites of ACE including ASN277, LYS454, PRO508 and PRO519 which do not all belong to the key active sites (sites S1, S1′ and S2) of ACE. Therefore, APKIEEV inhibits ACE activity with a complex modality of uncompetitive and noncompetitive inhibition [21], which is consistent with the results as shown in Figure 4B. As shown in Table 2, there are four short hydrogen bonds through which APKIEEV can bind to ACE. The T-score is one of the key indicators to evaluate the docking capability of ligand and ACE, and the required thrust value of T-score is 6.0 [20]. The T-score of APKIEEV is 11.33 (Table 2), suggesting that the combination between APKIEEV and ACE is strong [9]. This is also the main reason why APKIEEV has relatively high ACE-inhibitory activity (550.41 μmol/L, Figure 4A). It has been demonstrated that uncompetitive inhibitors usually showed lower ACE-inhibitory activity than that of competitive inhibitors [16]. Therefore, APKIEEV (an uncompetitive inhibitor) showed a lower ACE inhibition activity than that of peptide NMAINPSKENLCSTFCK (a competitive inhibitor of ACE) [18].

### 2.5. Antihypertensive Effect of Synthetic Peptides

**Figure 6:**
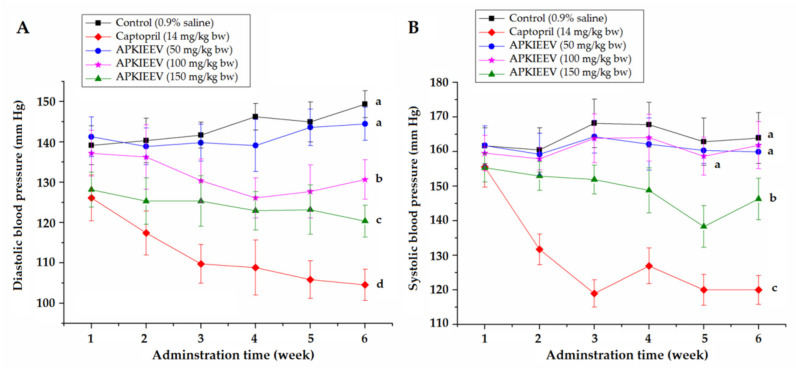
Effects of oral administration of individual peptide APKIEEV on systolic blood pressure (**A**) and diastolic blood pressure (**B**) of spontaneously hypertensive rats (SHRs). SHRs in the low-, middle- and high-dose group were orally administered the peptide at 50, 100 and 150 mg/kg/bodyweight once daily, respectively. SHRs of the positive control group were given captopril at 14 mg/kg/body weight once daily, whereas the SHRs in control group were just given physiological saline (0.5 mL). Different lowercase letters (a–d) near the lines mean significant difference (*p* < 0.05).

Antihypertensive effect of APKIEEV on spontaneously hypertensive rats (SHRs) are shown in Figure 6.

The oral administration of APKIEEV (at 100–150 mg/kg body weight) obviously (*p* < 0.05) decreased DBP of the SHRs (Figure 6A); whereas oral administration of APKIEEV at 150 mg/kg body weight significantly reduced systolic blood pressure of SHRs (*p* < 0.05) (Figure 6B), demonstrating that APKIEEV with dose of 150 mg/kg body weight exhibited antihypertensive effect in vivo. In contrast, APKIEEV with dose of 50 mg/kg body weight has no lowering effect on neither DBP nor SBP (*p* > 0.05). These results suggested that APKIEEV is a dose-dependent antihypertensive peptide. SHRs in the positive group that were given Captopril exhibited the lowest SBP and DBP among all the test groups throughout the experiment period, mainly attributed to the high ACE-inhibitory activity of captopril (IC_50_: 0.023 μM) [20]. Previous studies demonstrated that some ACE-inhibitory peptides did not exhibit antihypertensive effect in vivo, which is possibly the reason that their amino acid sequence can be destroyed when they are adsorbed in vivo [8,10]. Thus, the antihypertensive effect in vivo of ACE-inhibitory peptide should be determined. Moreover, APKIEEV at 150 mg/kg body weight showed a higher antihypertensive effect than that of peptide ADVFQPR identified from palm kernel glultelin-2 hydrolysates [5]. In addition, compared with SHRs in the negative control group, the body weight of SHRs in low-, middle-, and high-dose groups were not obviously different (Supplementary Table S1) (*p* > 0.05), demonstrating that oral administration of APKIEEV has no obvious side effect on weight of SHRs. However, the side effect of APKIEEV in vivo need to be done in further works.

### 2.6. Effects on Intracellular Endothelin-1 (ET-1)

**Figure 7:**
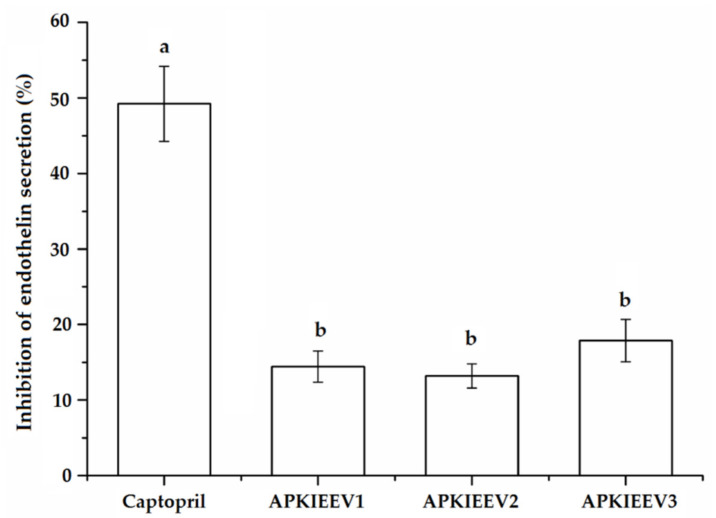
Effect of peptide APKIEEV on endothelin-1 secretion with Captopril (1 mg/mL) as positive control. APKIEEV1: APKIEEV at concentration of 0.5 mg/mL; APKIEEV2: APKIEEV at concentration of 1.0 mg/mL; APKIEEV3: APKIEEV at concentration of 1.5 mg/mL. Different lowercase letters on the bars (a–b) mean significant difference (*p* < 0.05).

ET-1 existing in the endothelin system is a vasoconstrictor, its excessive expression can increase blood pressure and cause cardiovascular disorders including coronary disease [4]. As shown in Figure 7, APKIEEV at all concentrations (0.5–1.5 mg/mL) demonstrated an inhibiting ability on expression of ET-1 (14.32–17.45%), but the inhibitory effect is not dose-dependent. The inhibition of APKIEEV is much lower than that of captopril (*p* < 0.05), but higher than that of the peptide VVLK identified in palm kernel expeller glutlin-2 and Antarctic krill protein [4,5]. Moreover, the results in Figure 4A, Figure 6 and Figure 7 demonstrate that APKIEEV can exhibit an antihypertensive effect in vivo through inhibiting ACE activity and/or suppressing the expression of ET-1.

## 3. Materials and Methods

### 3.1. Materials

Areca nut kernel was purchased from Sanpo Palm Ground Part Co., Haikou, China. Cellulase (from *Trichoderma viride G*, 3.0 × 10^5^ U), trypsin (5 × 10^4^ U/g) and alcalase (1 × 10^5^ U/g) were purchased from Tian Keji Co., Ltd. (Tianjin, China). EA.hy926 cells were purchased from the Type Culture Collection of the Chinese Academy of Sciences, Shanghai, China. Captopril, ACE (from rabbit lung) and N-Hippuryl-His-Leu hydrate (HHL) were purchased from Sigma Co., St. Louis, MO, USA. Endothelin-1 Elisa kit was from Pensui Biotech Co. (Wuhan, China). DMEM (Dulbecco’s modified Eagle’s medium) were obtained from JiQing Biotech. Co., Ltd. (Nanjing, China). Other chemicals and reagents were of analytical grade and purchased from Peisu Co., Ltd. (Xi’an, China).

### 3.2. Preparation of Areca Nut Kernel Globulin

Areca nut kernel globulin (ANKG) was prepared following the method of Li et al. [22] with some modifications. Briefly, areca nut kernel was dried at 45 °C for 16 h and ground to passed through a sieve of 0.2 mm mesh and then defatted three times using N-hexane. The defatted areca nut kernel was suspended in NaCl solution (0.2 mol/L, 1: 20, g/mL). The suspension was adjusted to pH 4.5 with 0.1 M HCl and cellulase (1 g/100 g ANKG) was added. The mixture was incubated at 50 °C for 2.5 h and then adjusted to pH 7.4 and stirred at 35 °C for another 2 h. After filtration, the filter was collected and residue was suspended in NaCl solution (0.2 mol/L, 1: 20, g/mL) again. This extraction progress was repeated in three times. The filtrates were pooled and centrifuged at 10,000× g for 25 min. The supernatant was collected and dialyzed against distilled water with a 3500 Da MWCO dialysis membrane (Solarbio Co., Tianjin, China) at 4 °C for 48 h. During the dialysis, the distilled water was changed at 2 h intervals until all globulin was precipitated. Then the dialyzed solution was centrifuged at 10,000× *g* for 12 min, the precipitate was collected, lyophilized and named as areca nut kernel globulin.

### 3.3. Preparation of Areca Nut Kernel Globulin Hydrolysate

Areca nut kernel globulin hydrolysate (ANKGH) was prepared following the same procedure as described by Zheng et al. [5]. Briefly, the ANKG (2 g) were re-dispersed in 100 mL of phosphate buffer (0.1 mol/L, pH 7.4) and mixed with alcalase (100 U/g ANKG). The reaction mixture was stirred at 45 °C for 120 min, and then adjusted to pH 7.0 and trypsin (100 U/g ANKG) was added. After incubation in a stirring water bath at 37 °C for 3 h, the mixture was incubated at 100 °C for 10 min. The mixture was centrifuged at 12,000× *g* for 15 min. The supernatant was collected and lyophilized with a dryer (FD-1C-50, Yancheng, China) to obtain ANKGH powder. The trinitrobenzenesulfonic acid method was used to determine the hydrolysis degree (DH) of ANKGH [23].

### 3.4. ACE Inhibitory Activity and Inhibition Kinetics

ACE-inhibitory activity was determined using the same procedure as described by Jimsheena and Gowda [24]. The concentration of the sample required for inhibiting 50% of ACE activity was defined as IC_50_ value. The ACE inhibition kinetics of peptides identified in ANKGH was analyzed by Lineweaver-Burk plots of 1/V versus 1/HHL in the presence of the inhibitor [17]. The concentrations of HHL (N-hippuryl-His-Leu hydrate) used were 0.76 to 7.60 mM.

### 3.5. Purification of ACE-Inhibitory Peptides from ANKGH

As per the method of Zheng, Zhang and San [25], ANKGH solutions (2 mg/mL) were pushed through an ultrafiltration membrane (0.22 μm, Jinteng Co., Tianjin, China). The filtrate was collected and separated with a Sephadex G-15 gel column (Φ16 × 800 mm, Huxi Co., Shanghai, China). The eluate was distilled water, the elution rate was 2.4 mL/min and the absorbance was determined at 220 nm. Each effluent fraction was pooled, lyophilized and used to ACE-inhibitory activity determination. The fraction showing the highest activity was chosen to purification on RP-HPLC system (SHIMADZU LC-20A) coupled with a Kromasil 100–5 C_18_ column (4.6 × 250 mm, 5 μm, Akzo Nobel N.V., Arlöv, Sweden). Distilled water was used as mobile phase A and the mobile phase B was a linear gradient of acetonitrile (5–35%, in 0.5 h) with a flow rate of 1.2 mL/min. The absorbance was measured at 220 nm. The absorbance was measured at 220 nm. The ACE-inhibitory activity of each subfraction was determined. The subfraction with the highest activity was subjected to amino acid sequence identification.

### 3.6. Amino Acid Sequence Identification

An Ultimate 3000 Series Ultra-High Performance Liquid System (UPLC, Thermo Scientific, Waltham, MA, USA) coupled with an InfinityLab Poroshell 120 EC-C_18_ column (10 × 0.21 cm, 1.9 μm, Agilent Technologies, Palo Alto, CA, USA) was used to further purify the closed subfraction. The UPLC system was carried out using ultrapure water as eluent B and gradient of acetonitrile (containing 0.1% formic acid) as eluent A at a flow rate of 0.25 mL/min. The gradient for eluent A was 0–2.5 min, hold at 5%; 2.6–12.5 min, at 10%; 12.6–20 min, at 5%; 20–22 min, at 52.5%; 23–30 min, at 95.0%; and 31–35 min, at 5%. The peptide was automatically selected on basis of fragment information from the electrospray ionization mass spectrometry (ESI-MS) analysis which was performed on a Q Exactive hybrid quadrupole-orbitrap mass spectrometer (Thermo Fisher, Bremen, Germany) with mass range of 120–1800 *m*/*z*, full MS 35,000, ddMS^2^ 17,500, and AGC target value of 1 e^5^. A De Novo™ software (Peak Studio 7.5, Bioinformatics Solutions, Inc., Waterloo, Ontario, Canada) was used to process the peptide sequence and molecular weight of the MS data [20].

### 3.7. In Silico Screening and Synthesis of Peptides

The database AHTPDB (http://crdd.osdd.net/raghava/ahtpdb/, accessed on 27 May 2021) and BIOPEP (http://www.uwm.edu.pl/biochemia/index.php/en/biopep/, accessed on 27 May 2021) were used to predict the antihypertensive activity and ACE-inhibitory activity of peptides identified in ANKGH, respectively [16,26]. Peptides should be accepted if the vector machine software scores (SVMS) are above zero or the average local confidence (ALC) is greater than 85% [27]. Some physicochemical properties including hydrophilicity, steric hindrance, amphiphilicity and isoelectric point (PI) of the peptides were also predicted by the database AHTPDB. After which, the peptide selected (the purity was more than 98%) were synthesized using a standard solid phase method by Danguang Biotech Ltd. Co. (Dalian, China).

### 3.8. Molecular Docking

The combination pattern between ACE and the peptide sequence identified in ANKGH was studied using the WYBYL-X2.11 software following the method of Ling, Sun, and Zhuang [20]. Here, the three-dimensional structure of ACE (PDB: 1O8A) was obtained from the Protein Data Bank (http://www.rcsb.org/pdb/home/home.do/, accessed on 27 May 2021). The T-scores as well as C-scores, number of hydrogen bonds and distance were calculated and used to evaluate the docking pattern between the peptide and ACE.

### 3.9. Antihypertensive Effect In Vivo

The antihypertensive effect of the synthesized peptide was determined as per the same procedure described by Zheng et al. [5]. Briefly, spontaneously hypertensive rats (SHRs, male, 12 weeks age) were purchased from VRL Animal Technology Co., Ltd. (Beijing, China). The SHRs were randomly divided into positive control group, low-, middle- and high-dosage groups, and negative control group. Each group contains four rats. SHRs in the high-, middle- and low-dosage groups were orally administrated with the synthesized peptide at 50, 100 and 150 mg/kg/bodyweight once daily, respectively; whereas, captopril (14 mg/kg/bodyweight) was used as the positive control. In addition, SHRs in the negative group were orally given saline (0.9%). After an adaptation period of one week, the weight, systolic blood pressure (SBP) and diastolic blood pressure (DBP) of SHRs were measured using the tail-cuff method once week [28]. Moreover, the animal experiment was conducted in conformity with institutional guidelines for the care and use of laboratory animals in Shanxi University of Chinese Medicine, Taiyuan, China. All the rats received human care conforming to the National Institutes of Health Guide for care and Use of Laboratory Animals (Publication No. 85-23, revised 1985).

### 3.10. Effects on Intracellular Endothelin-1

The effect of the synthesized peptide (APKIEEV) on intracellular endothelin-1 (ET-1) was demonstrated using the same procedure described by Zheng et al. [5]. Briefly, EA.hy926 cells were cultured in DMEM at 37 °C for 24 h in a humidified atmosphere with 5% CO_2_. The DMEM contains streptomycin (100 μg/mL), fetal bovine serum (100 μg/mL) and non-essential amino acid (10 mg/mL). The cells were transferred into 96-well plates and cultured in DMEM at 37 °C for 24 h, and then subjected to treatment of the synthesized peptide with different concentrations (0.5, 1.0 and 1.5 mg/mL) for 48 h; while, captopril (1 mg/mL) was used as the comparison. The growth medium in each well was collected and the ET-1 content was determined using an ET-1 Elisa kit.

### 3.11. Statistical Analysis

SPSS 20.0 (SPSS Inc., Chicago, IL, USA) was used for statistical analysis, results were expressed as the mean ± standard deviations (*n* ≥ 3), and Duncan’s multiple range test was used to variance analysis (*p* < 0.05).

## 4. Conclusions

A novel ACE-inhibitory heptapeptide (APKIEEV) was identified in areca nut kernel globulin hydrolysates. APKIEEV has high ACE-inhibitory activity (IC_50_: 550.41 μmol/L) and can combine with four active sites (not the key active sites) of ACE via five short hydrogen bonds. Moreover, dosage of APKIEEV at 150 mg/kg/bodyweight once daily exhibited a significantly lowering effect on the systolic blood pressure and diastolic blood pressure of SHRs and had considerable suppressing ability on intracellular endothelin-1. However, the bioavailability and action mechanism of APKIEEV need to be studied in the next work.

## Figures and Tables

**Table 1:** Amino acid sequence, predicted antihypertensive and ACE-inhibitory activity, and physicochemical properties of the peptides identified in areca nut kernel globulin hydrolysates by UPLC-ESI-MS/MS and screening in silico.

Peptide	Mass (Da)	SVMS	Prediction	ALC(%)	Hydrophilicity	Amphiphilicity	Hydrophobicity	Steric Hindrance	Isoelectric Point	IC_50_ (μmol/L)
EVDEVLNA	888.04	−0.47	Non-AHT	80	−0.09	0.32	0.49	0.74	3.58	ND
APKIEEV	784.99	0.77	AHT	92	0.74	0.89	−0.13	0.67	4.54	550.41
AFSKI	564.73	−0.78	Non-AHT	79	−0.30	0.73	0.05	0.69	9.11	ND
QFLMQIIRT	1149.53	−0.79	Non-AHT	75	−0.69	0.05	−0.09	0.78	10.11	ND
ALSSAGLQNQIK	1229.55	−1.10	Non-AHT	71	−0.18	0.51	−0.10	0.66	9.11	ND
ASGNSQGT	720.80	−0.47	Non-AHT	76	0.01	0.06	−0.18	0.44	5.88	ND
ENIDSSR	819.90	−2.22	Non-AHT	83	1.14	0.53	−0.50	0.71	4.38	ND

SVMS: vector machine software score; and AHT: antihypertension; and physicochemical properties were predicted using the AHTPDB database. ALC, average local confidence calculated using published data in BIOPEP database.

**Table 2:** Docking scores and hydrogen bonds observed between peptide APKIEEV and ACE from the molecular docking simulation.

Ligand	T-Score	C-Score	Hydrogen Bonds Number	Distance (Å)
APKIEEV	11.33	400	4	ASN277: 1.43; LYS454: 2.04; PRO508: 1.49, 2.39; PRO519: 2.01

## Supplementary Materials

The following are available online, Table S1: Body weight (g) of the spontaneous hypertensive rats administrated with peptide APKIEEV, Table S2: Heart rate of the spontaneous hypertensive rats administrated with peptide APKIEEV.

## Sample Availability

Samples of the areca nut kernel are available from the authors.
