# Supplementary material for: Identification, Characterization and Antihypertensive Effect In Vivo of a Novel ACE-Inhibitory Heptapeptide from Defatted Areca Nut Kernel Globulin Hydrolysates
Source: Molecules. 2021 May 31;26(11):3308. doi: 10.3390/molecules26113308 (PMC8199471; doi:10.3390/molecules26113308)
Supplement: Supplementary file 1 [file molecules-26-03308-s001.zip › molecules-1157715-Supplementary Material.pdf]

Supplementary Table S1 Body weight (g) of the spontaneous hypertensive rats administrated with peptide APKIEEV [a]

Time	Negative control				Captopril control				Middle dose group				High dose group				Low dose group			
0	255.2	252.2	248.3	238.6	274.4	249.3	239.1	231.1	269.3	263.6	258.5	233.7	265.8	252.2	242	236.9	249.4	245.6	237.2	259.6
1st	283.5	266.2	278.1	264.8	277.2	267.6	258.4	247.7	282.5	278.1	273.8	242.8	280.4	269.4	271.7	266	275.3	259.4	258.5	287.7
2nd	297.9	278.1	293.6	281.6	288.8	277.3	274.4	258.4	295.2	288.3	287.1	253.6	287.8	285.7	295.6	263.6	292	266.2	269.8	300
3rd	316.3	300.3	306.2	298.7	309.3	285	282.4	268.2	304	297.3	302.1	263.9	317.9	307.8	290.8	285	294	271.5	277.2	309.6
4th	322.9	313	316	305.4	317.4	289.4	291.2	287.3	314.9	319.2	300.6	276.3	323.7	316.4	302	293.6	314.7	297	288.8	317.1
5th	339	324.1	336.8	315.2	320.3	293.7	297.7	280.3	325.5	328.8	312.8	282.3	327.7	323.8	304.8	305.8	333	304.4	295.8	323.9
6th	342.1	331.2	343.2	319.2	329.6	302.5	313.8	292.6	332.6	332.7	325.8	291.7	347.5	344.7	324.5	320.2	339.8	312.3	312.4	331.6

[a] Rats in Low-, middle- and high- dose groups were orally given the peptide at 50, 100 and 150 mg/kg/bodyweight once daily, respectively.

Supplementary Table S2 Heart rate of the spontaneous hypertensive rats administrated with peptide APKIEEV [a]

Groups	Heart rate (bpm)																
**The first week**																	
Low dose group	149.4	121.3	139.9	144.5	149.4	139.9	115.6	138.7	142.4	142.4	132.6	142.9					
Middle dose group	123.2	125.2	134.6	142.5	146.9	140.4	111.3	131.9	156.2	134.7	127.9	123.3					
High dose group	117	129.1	129.3	140.5	144.4	140.9	117	125.1	140	127	123.2	113.7	121.2	122.8	131	117	129.1
Captopril control	5.51	7.12	6	5.81	6.8	6.95	7.52	6.65	6.14	7.41	6.57	6.49					
Negative control	5.8	5.87	6.78	6.69	6.92	6.41	6.43	6.45	6.67	7.92	8.22	6.67	5.83	7.59	6.77		
**The second week**																	
Low dose group	135.9	114.5	140.4	129.9	131.2	131.7	149.6	130.3	141.7	133.3	123.1	132.8					
Middle dose group	145.2	109.7	139.5	139.7	136.5	131.6	120.2	118.4	141.1	140.7	130.4	122.1	122.2				
High dose group	126.6	139.3	131.3	110.1	125.9	131.8	129	142.2	122.3	125.9	115.8	103.5		122.8	113.4	131.6	140.7

Captopril control	7.24	8.28	8.42	7.63	6.9	7	9.12	6.19	9.25	8.43	11.35	9.81						
Negative control	6.45	6.93	6.74	6.78	6.45	10.52	7.3	5.82	6.68	7.48	7.37	7.82	8.19	7.29	6.57	6.81	8.63	8.71
**The third week**																		
Low dose group	128.4	132.8	134.7	144.2	140.4	140.1	120.5	118.1	127.4	128.9	118.1	119.4	134.5					
Middle dose group	120.6	123.1	115.4	103.4	130.5	138.3	132.8	144.7	112.3	119.5	121.3	126.1	131.3	119.1				
High dose group	126.2	122.5	122.4	125	140.3	141.9	108.2	101.5	132.5	118.3	114.9	112.7	107.7	119.1	112			
Captopril control	6.92	5.91	5.61	7.28	7.91	7.18	6.11	6.04	6.27	7.53	7.29	7.63	6.48					
Negative control	8.1	7.08	8.19	6.19	6.34	7.14	8.22	6.28	7.61	8.57	8.64	8.6	6.97					
**The fourth week**																		
Low dose group	100.9	97.9	92.6	108.5	108.4	108.4	122.9	92.3	92.2	116.6	100.6	127.9	104.7	98.6	106.8	138.3	132.8	
Middle dose group	140.8	137.2	147.2	136.3	95.4	96.5	104	150.4	146.8	164.1	124	121.4	116.1	132.5	131.8			
High dose group	126.6	126.1	129.4	131	113.4	108.9	123.8	141.8	141	139	121.1	121.7	115.4					
Captopril control	5.88	6.67	6.52	6.64	6	6.19	5.39	5.61	6.57	7.41	8.33	7.63	7.86					

Negative control	6.58	7.02	8.09	6.83	8	6.9	8.33	6.23	7.91	6.14	7.1	5.67	5.8	5.91			
**The fifth week**																	
Low dose group	167.4	161.6	161.9	163.9	153.3	125.7	138.7	144	149	163.5	160.1	161.4					
Middle dose group	150.8	153.9	151.8	160.1	146.7	163.1	165.9	158.6	148.5	150.3	161.2	153.6	167.2	155.9	153.7	153.5	139.7
High dose group	156.5	137.2	142.5	141.6	147.6	146.6	121.3	141.7	138.7	163.5	162.6	155.6					
Captopril control	5.53	5.75	6.39	5.94	5.93	5.69	6.51	6.92	6.74	6.78	7.27	5.51	5.5	5.92	6.06	6.22	6.98
Negative control	6.08	6.19	6.43	6.14	6.46	6.38	6.45	6.64	6.95	6.85	6.84	6.5	6.58	6.54	6.58		

[a] Rats in Low-, middle- and high- dose groups were orally given the peptide at 50, 100 and 150 mg/kg/bodyweight once daily, respectively.
